# Diode-pumped wavelength-switchable quasi-three-level Nd:LiLuF_4_ laser on the ^4^F_3/2_→^4^I_9/2_ transition

**DOI:** 10.1371/journal.pone.0336865

**Published:** 2025-11-19

**Authors:** Chu Chu, Zhenhua Du, Nguyentuan Anh, Yuzhao Li

**Affiliations:** 1 College of Science, National University of Defense Technology, Changsha, China; 2 School of Physical Science and Technology, Bohai University, Jinzhou, China; 3 School of Physics and Astronomy, Yunnan University, Kunming, China; Rutgers University Newark, UNITED STATES OF AMERICA

## Abstract

We first demonstrate a continuous-wave (CW) wavelength-switchable quasi-three-level Nd:LiLuF_4_ laser operating at ^4^F_3/2_ → ^4^I_9/2_ transition. By shifting the pump beam waist location in the Nd:LLF, switching between single-wavelength (SW) and dual-wavelength (DW) operation can be achieved. The maximum SW output power of 4.03 W at 905 nm was obtained with a slope efficiency of 27.9% relative to the absorbed pump power. Furthermore, DW operation at 905 and 909 nm yielded the highest total output power of 3.21 W. Additionally, SW emission at 909 nm also reached a maximum output power of 2.21 W. Since the switching between SW and DW laser emissions is realized without introducing additional intracavitary losses, the method proposed in this paper provides a new approach for achieving high-power solid-state lasers with wavelength-switchable operation.

## 1. Introduction

In laser systems, lasing emission predominantly occurs on the strongest transition line due to gain competition. To achieve lasing on weaker spectral lines, selective suppression of the dominant transition is essential. This is implemented by introducing wavelength-selective loss elements [1–3], which disrupt the gain advantage of the dominant transition and thereby enable oscillation on weaker spectral lines. Similarly, multi-wavelength lasers generated simultaneously within a single gain medium require incorporating intracavity loss elements to suppress gain competition between transition lines, such as birefringent filter [4–6], etalon [7–9], cavity mirror with specific coating [10–12] and utilization of thermal anisotropy of gain medium [13,14]. However, those approaches inevitably introduce substantial losses, which not only severely reduce conversion efficiency but also increase system complexity. To address this problem, we propose a novel approach that enables switching between SW- and DW operation by shifting the pump beam waist in the active medium, without introducing any additional loss elements.

**Fig 1:**
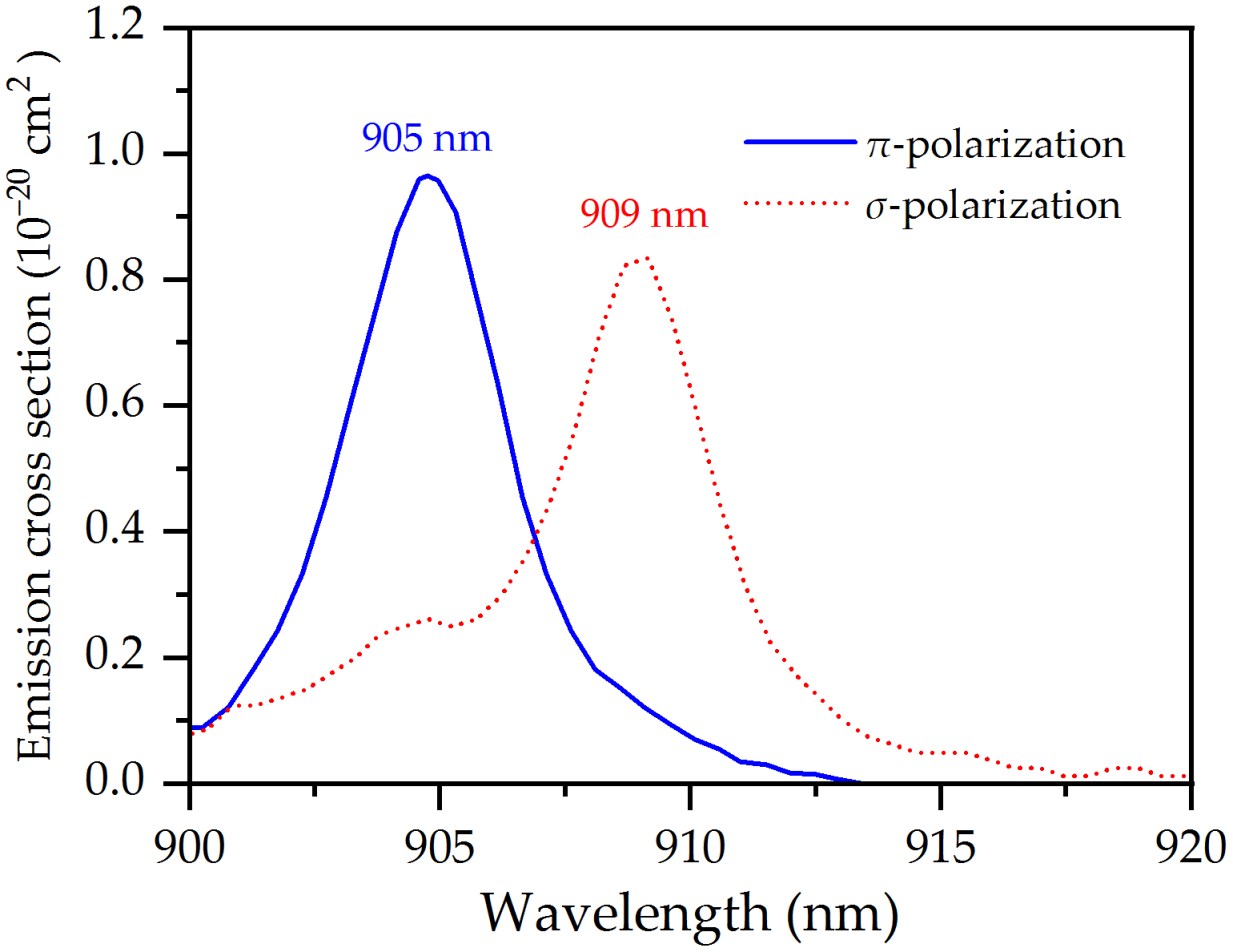
Emission spectra of the Nd:LLF in two polarized directions from 900 to 920 nm.

Nd:LiYF₄ (Nd:YLF) is a prominent solid-state laser material, valued for its intrinsic birefringence, extended upper-state lifetime, and low thermal impact [15–19]. Its structural analogue, Nd:LLF, exhibits superior properties including a greater fluorescence lifetime, a more pronounced thermally dependent lensing behavior, and marked spectral polarization anisotropy that facilitates polarized emission [20]. While laser operation of Nd:YLF covering the three transitions (^4^F_3/2_ → ^4^I_9/2_, ^4^F_3/2_ → ^4^I_11/2_ and ^4^F_3/2_ → ^4^I_13/2_) mentioned above is well established [21–23], the corresponding crystal Nd:LLF is formed by substituting Y³⁺ with Lu³ ⁺ ions. Successful laser demonstrations utilizing Nd:LLF have been achieved at 1.05 μm and 1.3 μm [20,24,25]. However, the generation of 0.9 μm laser radiation via the ^4^F_3/2_ → ^4^I_9/2_ transition in this host has not been published to date. The polarized emission characteristics of Nd:LLF at room temperature within the 900–920 nm range are displayed in Fig 1, as derived from the Füchtbauer-Ladenburg equation [26]. In this work, we proposed a design to obtain CW SW laser at 905 nm, DW laser at 905 and 909 nm and SW laser at 909 nm by using a low-doped and long Nd:LLF crystal. The SW and DW were switched by adjusting the waist location of the pump beam in the Nd:LLF crystal, not extra loss elements were inserted into the resonator. The maximum output power reached 4.03 W for the SW laser at 905 nm; 3.21 W for the DW laser operating simultaneously at 905 and 909 nm; and 2.21 W for the SW laser at 909 nm. This is the first to realize quasi-three level laser in the Nd:LLF crystal. Since no additional intracavity losses are introduced, our proposed method is expected to obtain high-power wavelength-switched lasers in other rare earth-doped crystals.

## 2. Experimental setup

**Fig 2:**
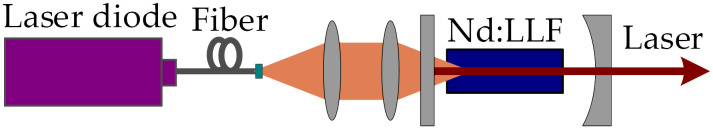
Experimental setup for the wavelength-switchable quasi-three-level Nd:LLF laser.

Fig 2 schematically depicts the wavelength-switchable quasi-three-level Nd:LLF laser. A 792 nm laser-diode (LD) with a maximum output power of 20 W was employed as the pump source. The pump light was delivered through a fiber with a core diameter of 400 μm and a numerical aperture (N.A.) of 0.22, focusing to a spot radius of 200 μm in the gain medium. The two identical plano-convex lenses (*L*_1_ and *L*_2_), each with a 300 mm focal length and anti-reflection (AR) coated at 792 nm, were used to collimate and focus the pump beam, respectively. This beam was directed into the gain medium, which was an *a*-cut Nd:LLF crystal with a 0.5 at.% doping concentration and a length of 15 mm. Both crystal facets were AR-coated for the 900–910 nm spectral range. The gain medium was first wrapped in indium foil and then secured within a red copper holder. This assembly was water-cooled to maintain a stable temperature of 20 °C. The cavity was formed by a plane input coupler (M_1_) and a plano-concave output coupler (M_2_). The M_1_ featured an HR coating at 900–910 nm and an AR coating at 792 nm, 1040–1060 nm, and 1310–1330 nm. The M2, with a −200 mm radius of curvature, had a transmittance of 2.7% at 900–910 nm. Ultimately, this output coupler was selected from several tested options (*T*_*oc*_ = 1.8%, 2.7%, 3.9%) for its superior results.

## 3. Results and discussion

For quasi-three-level lasers employing end-pumping, the absorbed pump power at threshold (*P*_*tha,i*_) can be described as [27,28]


$$P_{tha,i}=\frac{-ln\left(\text{1}-T_{oc}\right)+\text{2}{l_{c}\sigma_{abs,i}N}_{ion}{+L}_{i}}{\text{2}l_{c}{\tau_{i}\eta }_{i}}\frac{h\nu_{p}}{\sigma_{em,i}\tau_{i}}\frac{\text{1}}{J_{i}},$$ (1)

where the subscript *i* = 1 and 2 represents the 905 nm and 909 nm, respectively, *T*_*oc*_ is the output coupler transmittance, *l*_*c*_ is the Nd:LLF length, σ_*em*,*i*_ and σ_*abs*,*i*_ are the emission and absorption cross-sections at lasing wavelength, respectively, *N*_*ion*_ is Nd^3+^ ion density (cm^-3^), *L*_*i*_ is the cavity round trip loss, τ_*i*_ is the radiation lifetime of the upper level, *η*_*i*_ is the quantum efficiency, *h*ν_*p*_ is the pump photon energy, *J*_*i*_ is the overlapping integral between the pump and laser beams, which can be given by [29]


$$J_{i}=∭r_{p}(r,z)s_{i}(r,z)d\upsilon ,$$ (2)

where *r*_*p*_(*r*,*z*) is the normalized intensity distribution of the pump beam in the Nd:LLF crystal, and *s*_*i*_(*r,z*) is the normalized intensity distribution of the cavity mode for the lasing wavelength, and *r*_*p*_(*r*,*z*) and *s*_*i*_(*r,z*) can be written by [30], respectively,


$$r_{p}(r,z)=\frac{\alpha e^{-\alpha z}}{\pi \omega_{p}^{\text{2}}(z)(\text{1}-e^{-\alpha z})}\Theta \left[\omega_{p}^{\text{2}}(z)-r^{\text{2}}\right],$$ (3)

and


$$s_{i}(r,z)=\frac{\text{2}}{\pi \omega_{i}^{\text{2}}l_{c}}e^{\text{2}r^{\text{2}}/\omega_{i}^{\text{2}}},$$ (4)

where *α* is the absorption coefficient of the Nd:LLF crystal for the pump wavelength, *r* and *z* are radial and lateral coordinates respectively, and *ω*_*p*_(*z*) is the pump beam size in the Nd:LLF crystal, which can be given by [31]


$$\omega_{p}^{\text{2}}(z)=\omega_{p\text{0}}^{\text{2}}\left\{\text{1}+{\left[\frac{\lambda_{p}M_{p}^{\text{2}}}{{n\pi \omega }_{p\text{0}}^{\text{2}}}\left(z-z_{\text{0}}\right)\right]}^{\text{2}}\right\},$$ (5)

where *ω*_*p0*_, λ_*p*_, and *Μ*^2^ are the waist radius, wavelength and quality factor of the pump beam, respectively, *n*_*i*_ is the index of refraction of the Nd:LLF, *z*_0_ is the waist location the pump beam, *z*_0_ = 0 is taken at the incidence surface of the Nd:LLF. *ω*_0*i*_ is the radius of the laser spot, which is governed by the thermal focal length (*f*_*i*_) of the Nd:LLF, with its value derivable through ABCD matrix computations, and the *f*_*i*_ can be written as [32]


$$\frac{\text{1}}{f_{i}}=\frac{\xi_{i}P_{in}\alpha {\left(\frac{dn}{dT}\right)}_{i}}{{\pi K}_{c,i}}\int_{\text{0}}^{l_{c}}\frac{\text{exp}(-\alpha z)}{\omega_{p}^{\text{2}}(z)}dz,$$ (6)

where ξ _*i*_ is the heat conversion coefficient, *P*_*in*_ is the pump power incident at the Nd:LLF entrance face, (*dn*/*dT*)_*i*_ is the thermo-optic coefficient, *K*_*c,i*_ is the thermal conductivity. The parameters in the experiment: *T*_oc_ = 0.035, *l*_*c*_=15 mm, *σ*_*em,*1_ = 9.7 × 10^−21^ cm^2^, *σ*_*em,*2_ = 8.2 × 10^−21^ cm^2^, *σ*_*abs,*1_ = 0.2 × 10^−21^ cm^2^, *σ*_*abs,*2_ = 1.0 × 10^−21^ cm^2^, *N*_*ion*_=7.2 × 10^21^ cm^-3^ (0.5 at.% Nd^3+^), *L*_*i*_=0.01was measured using the Findlay-Clay method [33], *hν*_*p*_=2.51 × 10^-19^ J, *η*_1_=0.875, *η*_2_=0.871, *α*= 1.25 cm^-1^, *λ*_*p*_=792 nm, *M*^2^=50, *n* =1.45, *ξ*_1_=0.125, *ξ*_2_=0.129, (d*n*/d*T*)_1_ = −4.6 × 10^−6^ K^−1^, (d*n*/d*T*)_2_ =−8.8 × 10^−6^ K^−1^, *K*_*c*,1_ = 5.5 W/ m·K, and *K*_*c*,2_ = 3.4 W/m·K. The values of (d*n*/d*T*)_*i*_ and *K*_*c,i*_ here were measured by the method in Ref. [34]. With Eqs. (2)-(6) and the above parameters, the overlapping integrals between the pump and two laser beams were calculated as a function of the waist location of the pump beam for three different absorbed pump power, as shown in Fig 3. As observed in Fig 3, the overlapping integrals for both laser wavelengths were dependent not only on the waist position the pump beam but also on the pump power. This correlation arose because changes in pump power altered the thermal lensing focal lengths of the active medium in both polarized directions. Consequently, the beam spot sizes of the two laser wavelengths within the gain medium were modified, which in turn caused their overlapping integrals to be altered.

**Fig 3:**
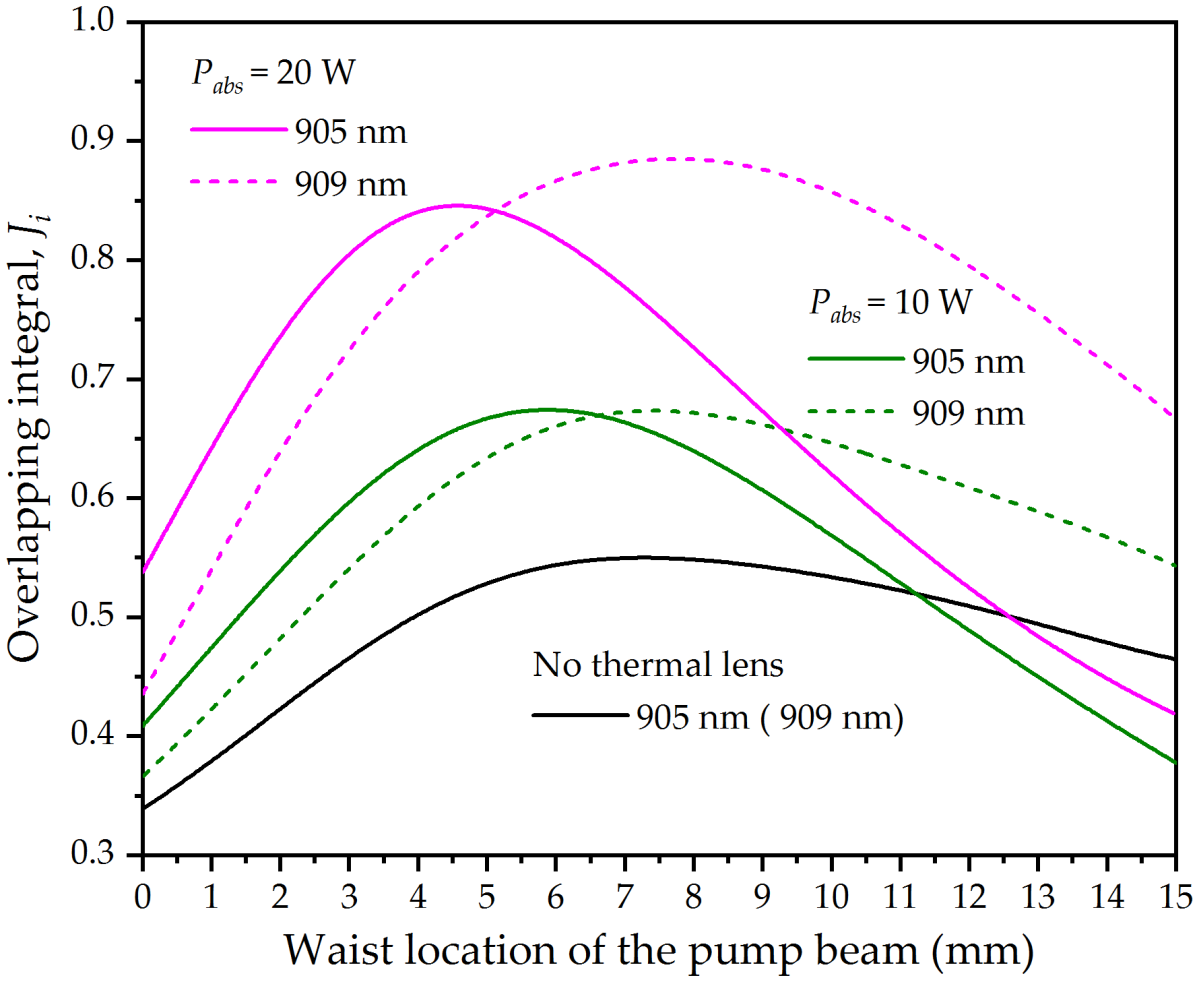
Overlapping integrals between the pump and laser beams versus the waist location of the pump beam for different absorbed pump power.

**Fig 4:**
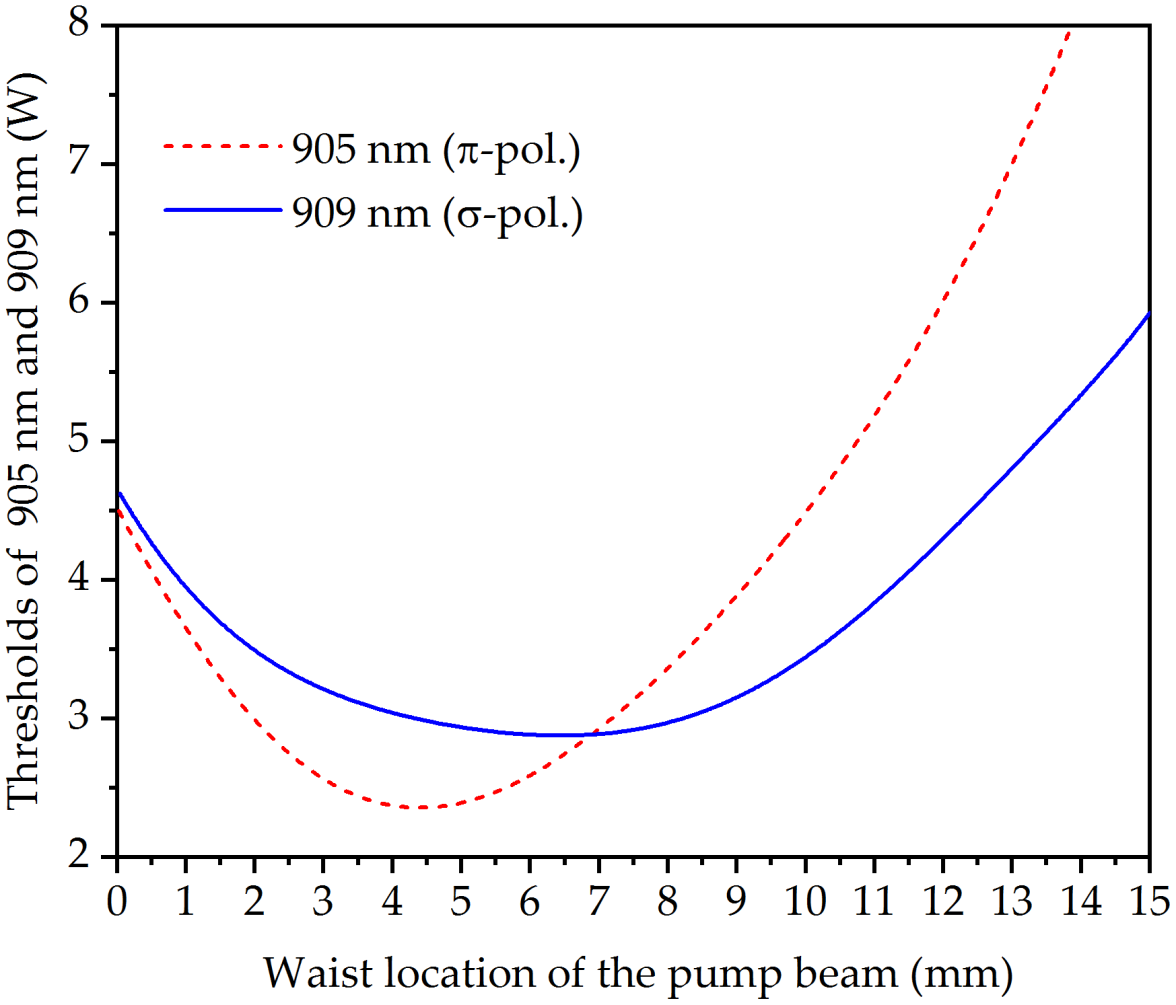
Simulated threshold powers of the 905 nm and 909 nm versus the pump beam waist location.

With Eqs. (1)-(6) and the relevant parameters, the thresholds (*P*_*abs,i*_) of the 905 nm and 909 nm were calculated as a function of the pump beam waist location, as shown in Fig 4. As shown in Fig 4, as the waist location of the pump beam moved from left to right, the 905 nm lasing threshold first monotonically decreased to 2.36 W and then monotonically increased beyond this point (*z*= 4.3 mm). On the other hand, the threshold power of the 909 nm wavelength reached a minimum at *z*= 6.5 mm and then monotonically increased. It was also found that the two curves intersected at *z* = 7.0 mm, indicating an equilibrium in the threshold power for the two lasing wavelengths. This means that at this point, 905 nm and 909 nm wavelengths can be generated simultaneously. Since *σ*_*em*,1_>*σ*_*em*,2_, *σ*_*abs*,1_≈*σ*_*abs*,2_ and *L*_1_≈*L*_2_, it can be concluded that *P*_*tha*,1_ < *P*_*tha*,2_, when there is no difference between the overlapping integrals *J*_1_ and *J*_2_. Compared with the 909 nm line, the 905 nm wavelength has a lower threshold (*P*_*tha*,1_ < *P*_*tha*,2_) and a larger emission cross-section (*σ*_*em*,1_>*σ*_*em*,2_), resulting in laser emission advantages at 905 nm. To obtain the DW operation, it is necessary to generate an appropriate difference between the *J*_1_ and *J*_2_ by moving the pump beam waist location to achieve the condition that *P*_*tha*,1_≥ *P*_*tha*,2_. The result of *P*_*tha*,1_> *P*_*tha*,2_ means that the laser will first emit the radiation at the weaker line at 909 nm and then simultaneously emit the radiation at 905 nm under a higher pump power. *P*_*tha*,1_> *P*_*tha*,2_ means that the weak 909 nm is emitted first, and then 905 nm and 909 nm wavelengths are emitted simultaneously at a higher pump power.

**Fig 5:**
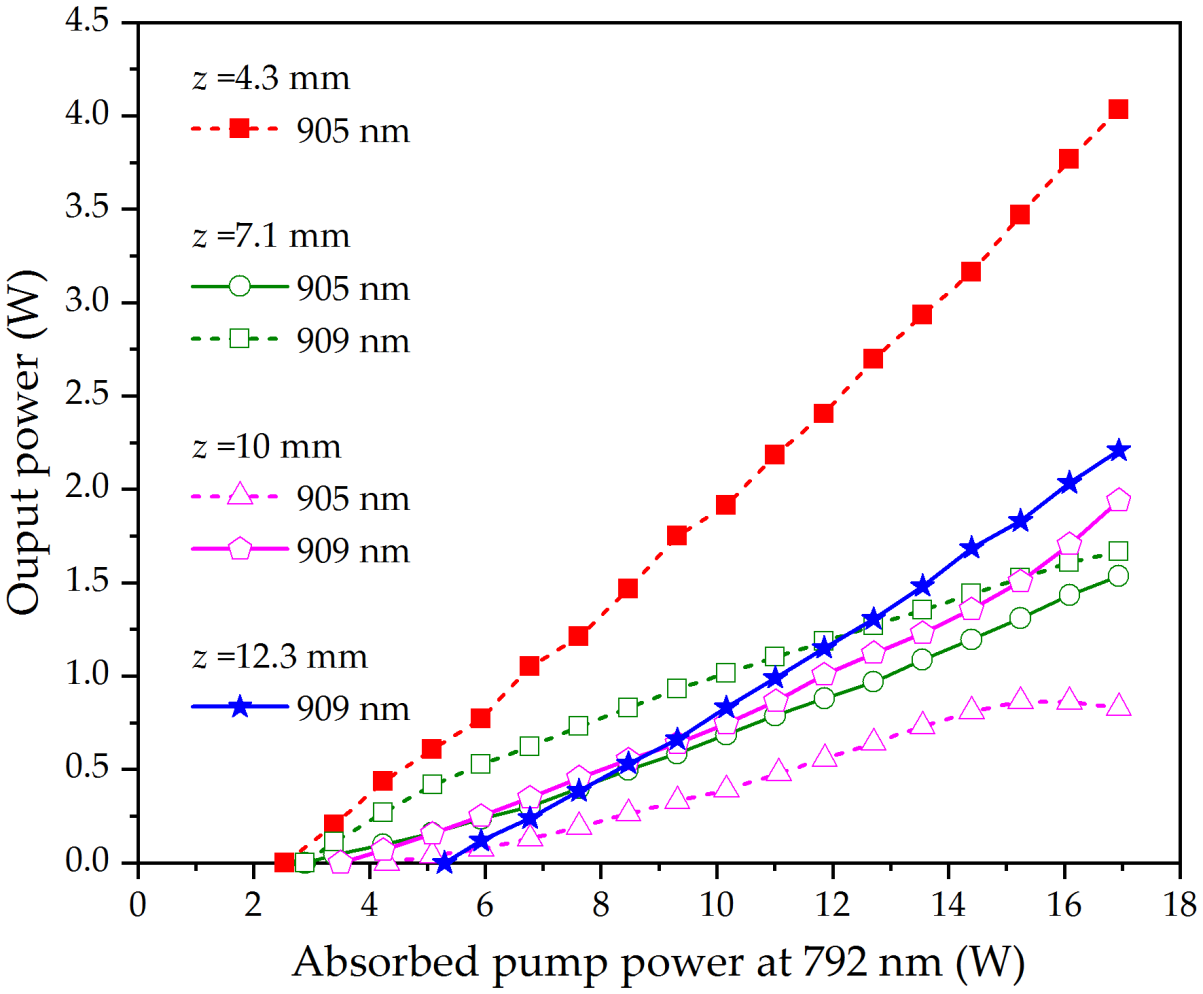
Output powers at 905 nm and 909 nm versus the absorbed pump power for different waist locations of the pump beam.

Fig 5 shows the dependence of the measured output powers on the absorbed pump power for different pump beam waist locations. By moving the pump beam waist location, we found that the laser threshold reached its minimum of 2.45 W when the pump beam waist was positioned at *z* = 4.3 mm. At an absorbed pump power of 16.94 W (corresponding to an incident pump power of 20 W), 4.03 W of 905 nm laser output was achieved, yielding a slope efficiency of 29.7% and an optical-to-optical conversion efficiency of 23.8%, both relative to absorbed pump power. Continue to move the pump beam waist to *z*= 7.1 mm, the DW laser at 905 and 909 nm with orthogonal polarization was obtained simultaneously. The threshold absorbed pump power of the DW laser was 2.92 W. The DW laser was separated into two beams with a polarizing beam splitter (PBS), and the output powers of 905 and 909 nm were measured separately. At an absorbed pump power of 16.94 W, the total power output of 3.21 W (1.67/1.54 W at 905/909 nm) was obtained. The corresponding total optical conversion efficiencies reached 18.9% relative to the absorbed pump power.

To better understand the operating characteristics of DW laser, the pump beam waist location was further moved to the right so that *P*_*tha*,1_> *P*_*tha*,2_. It can be found that the 909 nm wavelength was emitted first, and then the 905 nm wavelength was generated. We take *z*=10 mm to study the DW laser performance. The absorbed pump power at threshold was 3.51 W at 909 nm, and 4.32 W at 905 nm, which was basically consistent with the calculation result in Fig 4. The 909 nm output increases monotonically to its peak of 1.94 W at 16.94 W absorbed pump power. On the other hand, the 909 nm output rises linearly to a 0.87 W peak at 15.23 W absorbed pump power before monotonic decline. When the pump beam waist was moved to *z* = 12.3 mm, only the 909 nm wavelength was emitted. This occurs because as the pump beam waist approaches the right edge of the active medium, the difference in overlap integrals between the pump mode and the two laser modes increases significantly (see Fig 3), causing the 905 nm transition gain to dominate. The threshold absorbed pump power at 909 nm was 5.32 W. At an absorbed pump power of 16.94 W, an output power of 2.21 W was achieved, yielding a slope efficiency of 18.9% and an optical-to-optical conversion efficiency of 13.0%, both relative to absorbed pump power.

**Fig 6:**
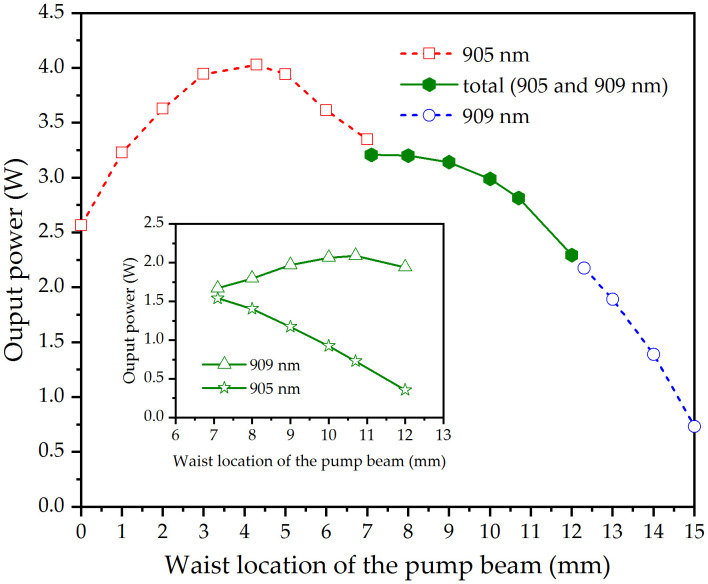
Output power of the emission wavelengths versus the pump beam waist location at the absorbed pump power of 16.94 W. Inset: the output power of the DW at 905 and 909 nm versus the pump beam waist location.


When the pump power was fixed at 16.94 W, the dependence of the output power of the quasi-three-level Nd:LLF laser on the pump beam waist location is shown in Fig 6. It can be seen in Fig 6 that as the pump beam waist moves from left to right, the 905 nm wavelength was generated first, and its power increased and then decreased. This phenomenon can be explained by the overlapping efficiency (*η*_*overlap*_,_*i*_) between the pump and laser beams, which can be expressed as [35]

$$\eta_{overlap,i}=\frac{{\left[∭r_{p}(r,z)s_{i}(r,z)d\upsilon \right]}^{\text{2}}}{∭r_{p}(r,z)s_{i}^{\text{2}}(r,z)d\upsilon }.$$ (7)

**Fig 7:**
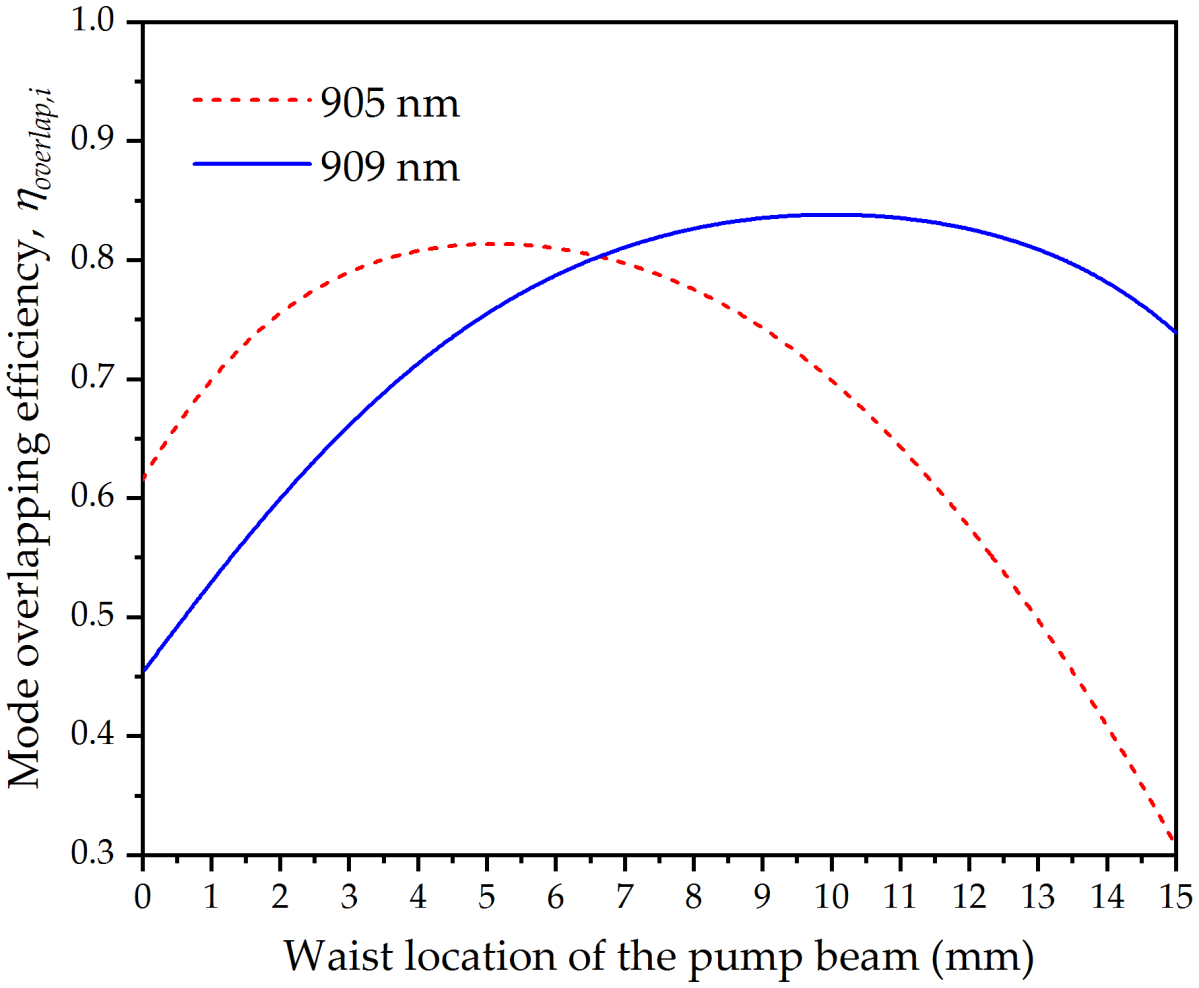
Mode overlapping efficiencies between the pump and laser beams versus the waist location of the pump beam at the absorbed pump power of 16.94 W.

Fig 7 presents the overlap efficiencies between the pump and laser beams, calculated using Eq. (1) as a function of the pump beam waist location. As shown in Fig 7, as the waist location of the pump beam moved from left to right, the overlap efficiency between the pump and 905 nm laser beams first monotonically increased and then monotonically decreased, which was consistent with the 905 nm output power in Fig 6.

When the pump beam waist moved beyond *z* = 7.1 mm, simultaneous DW emission at 905 nm and 909 nm began to occur. The inset in Fig 6 shows the dependence of the output power of each wavelength of the DW emission on the waist location of the pump beam. It can be seen that as the pump beam waist moves to the right, the output power of the 909 nm wavelength shows a trend of first increasing and then decreasing, while the output power of the 905 nm wavelength continuously decreases. This was consistent with the variation of the mode overlapping efficiencies between the pump and two laser beams in Fig 7. The decrease in the total output power was due to the weakened combined influence of the mode overlapping efficiency between the pump and two laser beams when the pump beam waist moves to the right. When the pump beam moved beyond *z* = 12.3 mm, only a wavelength of 909 nm was emitted. Beyond this point, as the pump beam waist moves further, the spatial mode matching efficiency between the 905 nm laser and pump beams decreases significantly. This causes severe mode mismatch between the pump and laser beams, thereby allowing the 909 nm wavelength to gain a relative advantage in gain competition.

**Fig 8:**
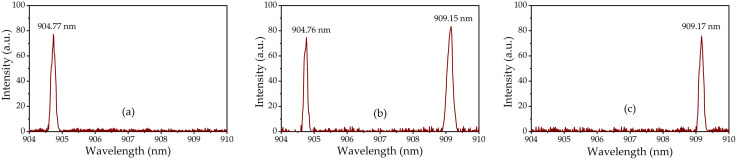
Laser spectra of the (a) π-polarized SW at 904 nm, (b) orthogonally polarized DW at 905 and 909 nm and (c) σ-polarized SW at 909 nm.

Using the LABRAM-UV spectrum analyzer (resolution: 0.01 nm) to scan the laser wavelengths and software to process the data, we obtained the laser spectra shown in Fig 8: π-polarized SW at 905 nm, orthogonally polarized DW at 905 and 909 nm, and σ-polarized SW at 909 nm emissions at maximum pumping. Their central wavelengths were 904.77 nm (π-polarized SW), 904.76 nm and 909.15 nm (orthogonally polarized DW), and 909.17 nm (σ-polarized SW), with corresponding full-width-at-half-maximum (FWHM) linewidths of 0.25 nm, 0.21 and 0.31 nm, and 0.27 nm, respectively.

The beam quality of the laser output at maximum power was measured using the knife-edge technique. The measured M² factors for the 905 nm (SW), 905 and 909 nm (DW), and 909 nm (SW) emissions were 1.06, 1.16 and 1.21, and 1.13, respectively. The power stabilities of the SW and DW lasers were measured with a with a Field-Master-GS power meter. The RMS power fluctuations over 1 hour at maximum output power were approximately 2.1% for 905 nm (SW), 2.6 and 3.4% for 905 and 909 nm (DW), and 2.5% for 909 nm (SW), respectively.

## 4. Conclusion

A diode-pumped CW wavelength-switchable quasi-three-level Nd:LLF laser on the ^4^F_3/2_ → ^4^I_9/2_ transition was demonstrated. By shifting the pump beam waist location in the active medium, switching between SW and DW operation can be achieved. The maximum SW output power of 4.03 W at 905 nm was obtained with a slope efficiency of 29.7% relative to the absorbed pump power. Furthermore, DW operation at 905 nm and 909 nm yielded the highest total output power of 3.21 W. Additionally, SW operation at 909 nm also reached a maximum output power of 2.21 W. The 905 nm laser, as the core light source for vehicle-mounted LiDAR ranging and industrial micro-trace detection, has both low atmospheric loss and high-resolution imaging characteristics. The 909 nm laser, with its deep tissue penetration and compatibility with polymer materials, dominates biomedical navigation and packaging defect detection. The 905 and 909nm dual-wavelength lasers, through differential absorption spectroscopy technology and millisecond-level dynamic switching capability, build an integrated platform for high-precision analysis of semiconductor doping and real-time dual-parameter imaging during surgery, becoming a cross-domain technology engine for high-end optoelectronic systems. Since the switching between SW and DW emissions is realized without introducing additional intracavitary losses, the method proposed in this paper provides a new approach for achieving high-power solid-state lasers with wavelength- switchable operation.
